# Multisite collaboration using REDCap to capture library data

**DOI:** 10.5195/jmla.2019.768

**Published:** 2019-10-01

**Authors:** Carrie Grinstead, Amanda Schwartz

**Affiliations:** Regional Medical Librarian, Providence St. Joseph Health, 501 South Buena Vista Street, Burbank, CA 91505, carrie.grinstead@providence.org; Library Specialist, Providence St. Joseph Health, 500 West Broadway, Missoula, MT 59802, amanda.steinvall@providence.org

## Abstract

In January 2018, library services at Providence St. Joseph Health merged to form a single, unified system, incorporating nine libraries and sixteen full-time staff. As a small, nonclinical team of librarians, we needed to make sure our work and value were visible to clinicians, administrators, and other nonlibrary stakeholders. Using REDCap, we developed a form to seamlessly collect statistics about our services.

Providence St. Joseph Health (PSJH) is a large, complex health organization, employing over one hundred thousand employees and encompassing fifty-one hospitals and eight legacy brands. In January 2018, library services at PSJH merged to form a single, unified system, incorporating nine libraries and sixteen full-time staff. As a small, nonclinical team of librarians, we needed to make sure our work and value were visible to clinicians, administrators, and other nonlibrary stakeholders.

Collecting and reporting library service statistics is critical in this integrated service environment to show the value and impact of services. We faced several challenges in collecting and reporting information about our services, including the disparate data collection methods of our legacy organizations, the variation in services offered at different libraries, and our large geographic spread. Our merged library serves patrons in seven states, and much of our work, including communication with patrons and each other, is conducted over email.

We began by assessing current collection practices regarding service statistics. One librarian compiled a list of items currently tracked at each hospital library. We chose REDCap, a secure web application for building and managing online surveys, early in our process. REDCap was easy to use and allowed us to build a web form that would be accessible on and off site, and regardless of legacy organization.

After selecting the tool, we convened a small task force to decide what information we would continue to collect and at what level of detail. This group included representatives from each legacy organization. We held several conference calls, then built a sample form, tested it for one month, and made necessary revisions. A sample of our completed form is available online: System Library Services Usage Statistics. This form is very similar to our tool but does not collect actual data.

We used our revised form to collect statistics for the final eight months of 2018. Each month, our librarian in Los Angeles developed a brief summary report that covered the entire system and included a cumulative counter of total requests. These reports were submitted to the library director and then to a system-level chief operating officer. At the end of the year, we reviewed our use of the form and made several adjustments for 2019. These adjustments included a new field, “Patient Experience,” documenting our contributions toward a system-level strategic initiative.

Our REDCap form facilitates simple, seamless data entry and fast reporting. A major advantage to the REDCap form is its flexibility: as we assessed our needs, we discovered that many of our core services were shared across sites. Other activities, including support for patients and families, are common at some locations and rare at others. We used REDCap’s branching logic functions to make certain fields appear only when they were relevant, which allowed us to collect a great deal of information while keeping the form short and user-friendly.

Information from our form was vital to creating several annual reports for 2018, including a narrative account of our services, an infographic, and several summary sheets for particular hospitals. We shared the summaries with hospital and system administrators and printed the infographic for display in all locations (Figure 1). These reports have garnered positive feedback from administrators, created greater understanding of the depth and breadth of library services, and contributed to a stable budget amidst a climate of increasing cuts.

**Figure 1:**
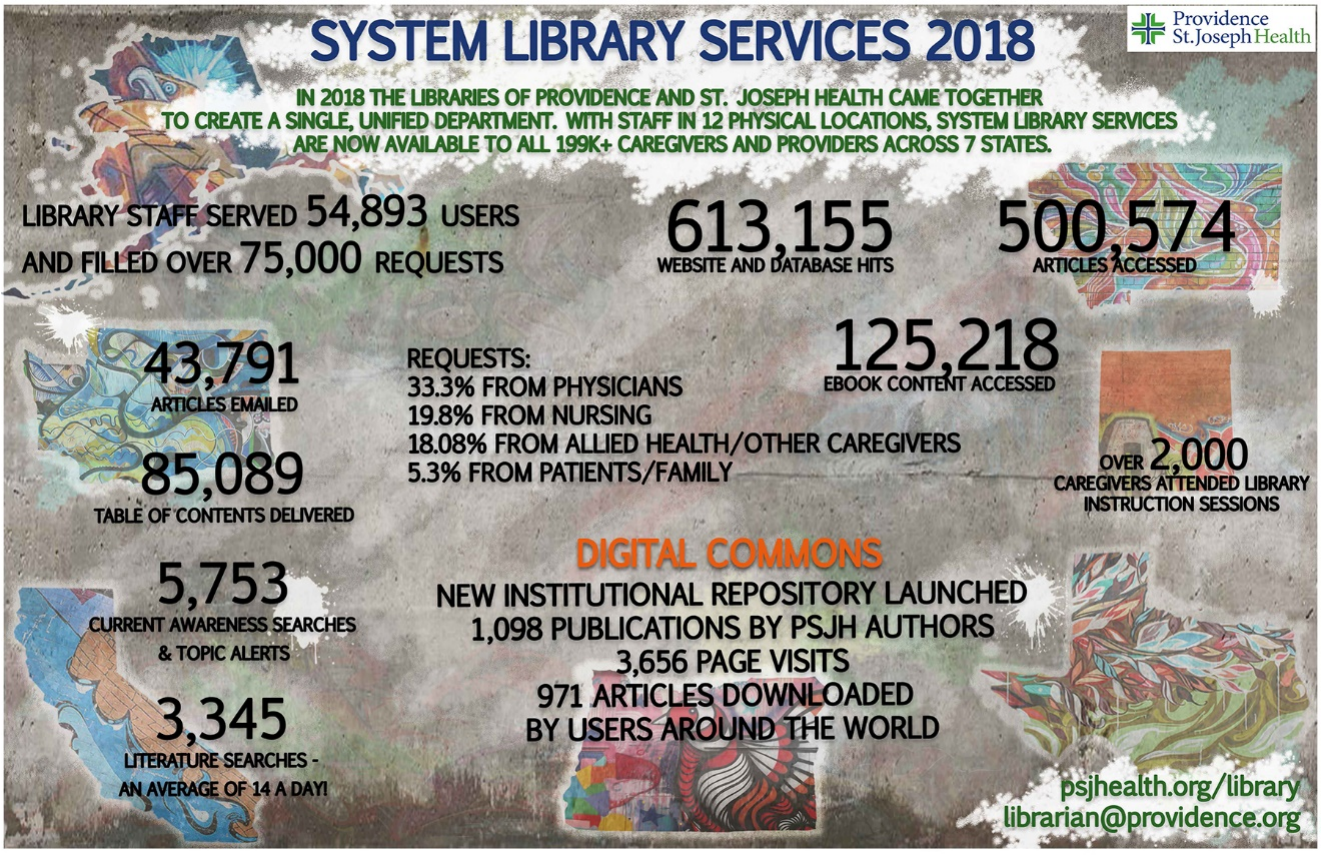
Infographic of Providence St. Joseph Health system library services in 2018

**Carrie Grinstead, MLIS, AHIP**, carrie.grinstead@providence.org, https://orcid.org/0000-0003-0400-7667, Regional Medical Librarian, Providence St. Joseph Health, 501 South Buena Vista Street, Burbank, CA 91505

**Amanda Schwartz**, amanda.steinvall@providence.org, https://orcid.org/0000-0002-1760-4308, Library Specialist, Providence St. Joseph Health, 500 West Broadway, Missoula, MT 59802

